# Psychometric properties of the World Health Organization WHOQOL-AGE Scale in Singapore

**DOI:** 10.1007/s10433-024-00803-3

**Published:** 2024-03-20

**Authors:** Rachael Zhi Yi Lee, Winson Fu Zun Yang, Rathi Mahendran, Lidia Suárez

**Affiliations:** 1 School of Social and Health Sciences, James Cook University, 149 Sims Drive, Singapore, 387380 Singapore; 2 Department of Psychological Science, Texas Tech University, 2700 18th St, Lubbock, TX USA; 3 Yeo Boon Khim, Mind Science Centre, National University of Singapore, 21 Lower Kent Ridge Rd, Singapore, 119077 Singapore; 4 Clarity Singapore Limited, Block 854 Yishun Road #01-3511, Singapore, 760854 Singapore; 5 Meditation Research Program, Department of Psychiatry, Massachusetts General Hospital, Harvard Medical School, Boston, MA USA; 6 Athinoula A. Martinos Center for Biomedical Imaging, Department of Radiology, Massachusetts General Hospital, Harvard Medical School, Boston, MA USA; 7 Mind Care Clinic @ SBF, 160 Robinson Road, #05-07 SBF Center, Singapore, 068914 Singapore

**Keywords:** Quality of life, WHOQOL-AGE, Asia, Psychometrics, Confirmatory factor analysis

## Abstract

WHOQOL-AGE is a promising quality of life (QOL) tool that has not been fully validated in Asia. The present study aimed to verify its factor structure and psychometric properties among community-dwelling older adults in Singapore. This study was cross-sectional and used data (*N* = 593) from the Community Health and Intergenerational study that interviewed older adults between 2018 and 2021. Confirmatory factor analysis (CFA) was used to examine the factor structure of the WHOQOL-AGE, and Cronbach’s alpha coefficients were employed to examine internal consistency. Spearman’s rho correlations coefficients between WHOQOL-AGE and other related scales (Satisfaction with Life and the Friendship) examined convergent validity. A Pearson’s correlation coefficient between WHOQOL-AGE and compassion scale examined discriminant validity. An independent *t* test was used to demonstrate known-groups validity, examining differences in QOL scores between individuals with and without chronic medical conditions. Findings supported a bifactor model with more satisfactory goodness-of-fit indices than the original two-factor model and the two-correlated factor model. WHOQOL-AGE showed adequate internal consistency (Cronbach’s alpha coefficients > .70). Good convergent validity was demonstrated by moderate-to-large correlations between WHOQOL-AGE and satisfaction with life (*r*_s_ = .54) as well as social connectedness (*r*_s_ = .33). Discriminant validity was shown by low correlations between WHOQOL-AGE and compassion (*r* = .19). Findings also indicated good known-groups validity (*p* < 0.01). The WHOQOL-AGE showed promising psychometric properties using an Asian convenience sample and can be useful in large-scale studies or busy clinical settings.

## Introduction

Owing to the advances in medicine and improved technology, many diseases can be treated effectively, resulting in decreased morbidity and mortality, and an increase in life expectancy worldwide (Bengtsson and Keilman 2019; Kyu et al. 2018). Countries continue to need population-based health data to adapt to the changing healthcare landscape by understanding their community public health problems, in order to plan and evaluate effective healthcare policies and treatment (Balogun et al. 2017; Hickey et al. 2010; Shrestha et al. 2015). Quality of Life (QOL) is a complex multidimensional concept that has been a target for research and practice worldwide (Bulamu et al. 2015; Haraldstad et al. 2019; Pequeno et al. 2020; The WHOQOL Group 1998). For instance, QOL instruments guide healthcare providers to make decisions about disease management (Llewellyn & Skevington 2016), provide policy makers and researchers with population-based data to inform policies (Fernandez-Mayoralas et al. 2012; Purba et al. 2018; National Council of Social Service 2017), and design preventive and rehabilitation programs (Aw et al. 2019; Chung et al. 2015).

According to the WHOQOL Group (1994), QOL is defined as “the individuals’ perception of their position in life in the context of the culture and value systems in which they live and in relation to their goals, expectations, standards and concerns.” Despite generic QOL measures having advantages such as allowing researchers to compare QOL between healthy and sick individuals, and across different social and cultural backgrounds, it has been argued that they may not capture areas of QOL relevant to older adults such as social participation, autonomy/independence, or safe living environment (Bowling et al. 2012; Gabriel & Bowling 2004; Kalfoss and Halvorsrud 2009; Power et al. 2005). Furthermore, older adults reported that it was their capability to achieve things or participate in valued activities that contributed to their QOL, while health served as a catalyst (Makai et al. 2014; Milte et al. 2014). As such, specific QOL instruments for older adults were developed such as the 43-item Elderly Quality of Life Index (Paschoal et al. 2008) and the 24-item WHOQOL—Older adults (WHOQOL-OLD; Power et al. 2005) that is used in conjunction with the 26-item Abbreviated World Health Organization Quality of Life questionnaire (WHOQOL-BREF; Skevington et al. 2004). However, their long administration time may pose a challenge for older adults to complete, especially when time is limited (e.g., clinical setting). Findings from a systematic review (Pequeno et al. 2020) indicated that research studies reported using easier and shorter measures such as the Medical Outcomes Study Short-Form 36/12 (SF-12/36; Ware et al. 1993) as compared to longer WHOQOL assessments. Hence, to address WHOQOL-OLD’s long administration time and SF-36/12’s lack of specific domains important to older adults, the WHOQOL-AGE was developed (Caballero et al. 2013).

**Fig. 1:**
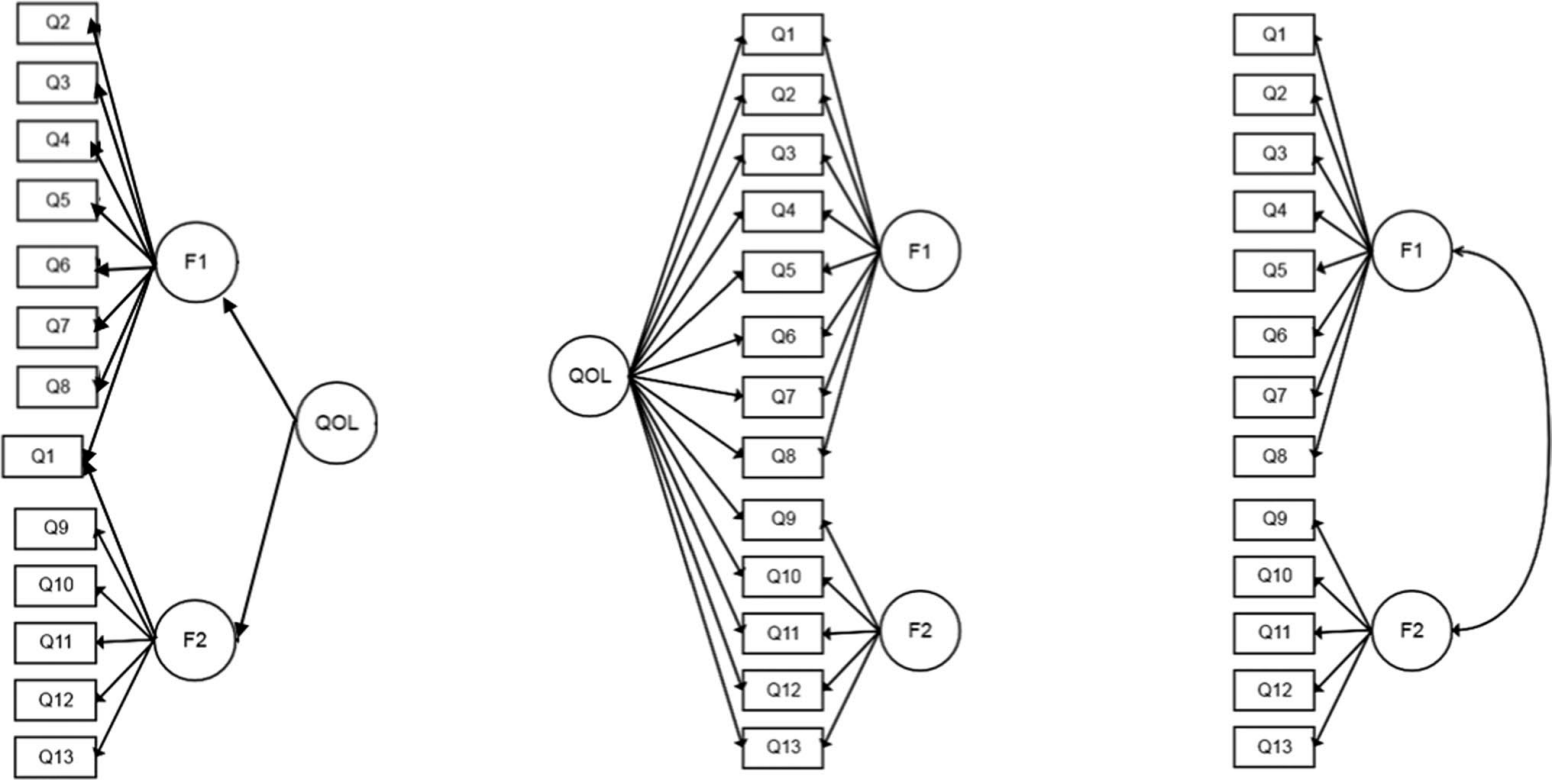
Graphic representation of proposed models for the WHOQOL-AGE. Note. This figure shows a two-factor model with cross-loading on item Q1 (left side) (Caballero et al. 2013), as well as a bifactor model (middle) and the two-correlated factor model (right side) (Lin et al. 2020; Santos et al. 2018)

The 13-item WHOQOL-AGE was derived from the EUROHIS-QOL 8-item index (Schmidt et al. 2006) and the WHOQOL-OLD short-form version 1 (Fang et al. 2012) to create a questionnaire that contained items from different domains relevant to older adults such as psychological, physical, environmental and social QOL, as well as capture their abilities in sensory abilities, social participation, autonomy, future activities and intimacy, which are all related to QOL. This short instrument was designed to be used in large-scale studies and busy clinical settings. The WHOQOL-AGE has been validated in countries such as Finland, Poland, Spain and Taiwan, showing good psychometric properties (Caballero et al. 2013; Lin et al. 2020; Santos et al. 2018; Özcan & Eser 2020), refer to Table 1. Findings confirmed a scale that comprised one second-order factor representing QOL and two first-order factors, where the overall QOL item 1 loaded on both factors (see left figure in Fig. 1). Results from the original study revealed a two-factor model with cross-loading on item Q1 and showed good internal consistency (Cronbach’s *α* = 0.91), strong convergent validity with satisfaction with life (*r* = 0.75) and discriminant validity with net affect validity (*r* = 0.35) as well as good known-groups validity between healthy (*M* = 74.19, *SD* = 13.21) and physical ill (*M* = 64.29, *SD* = 16.29) individuals (*p* < 0.001) (Caballero et al. 2013). The study by Santos further explored the multidimensionality of the WHOQOL-AGE and tested two structural models (Santos et al. 2018): a bifactor model and two-correlated factor model (see middle and right figures in Fig. 1). Findings provided evidence that the factor structure of the WHOQOL-AGE fitted better with a bifactor model and demonstrated partial invariance across three European countries. Although the WHOQOL-AGE showed partial invariance across three countries, there is a need to examine the scale structure for different cultures and populations. Moreover, in their analyses, the original model (Caballero et al. 2013) was not compared. Another study subsequently compared several factor structures of a translated WHOQOL-AGE among Taiwanese older adults and examined measurement invariance (Lin et al. 2020). Results favored the bifactor model found in a previous study conducted in Europe (Santos et al. 2018), whereby goodness-of-fit indices were the best among all the previously proposed models. In addition, WHOQOL-AGE was found to have measurement invariance across genders, educational levels, living settings and ages. This provided further evidence of a bifactor model underlying the scale in another cultural setting and population (Lin et al. 2020).

**Table 1:** Goodness-of-fit Indices and Psychometric Properties from Studies that Have Validated WHOQOL-AGE

Study	Sample characteristics	Factor structures (supported model)	Goodness-of-fit Values					Other psychometric properties measured
			RMSEA	CFI	TLI	SRMR	AIC	
Caballero et al. (2013)	Developmental (*N* = 6993) and Validation samples (*N* = 2994) from Finland, Poland and Spain; Older adults (≥ 50 years old) from noninstitutionalized setting	Two-factor model with cross-loading on item Q1 (original)	.077	.92	.90	**–**	–	Cronbach’s α (.91); convergent, discriminant, & known-groups validity
Santos et al. (2018)	Same sample (*N* = 9987) as Caballero et al. (2013)	Bifactor model (supported)	.054	.971	.952	**–**	266,646.879	Measurement invariance (between countries)
		Two-correlated factor model	.071	.936	.917		268,868.742	
Lin et al. (2020)	Convenience sample from Taiwan; older adults, ≥ 50 years old from community and institutionalzed settings (*N* = 522)	One-factor model	.029	.994	.993	.055	–	Measurement invariance (gender, education, setting); Cronbach’s α (.90); known-groups validity
		Two-correlated factor	.024	.996	.995	.052		
		Two-factor model	.025	.995	.994	.052		
		Bifactor Model (supported)	.000	1.000	1.009	.029		
Özcan and Eser (2020)	Sample from Turkey; older adults (*M*_age_ = 73.09) from urban and rural settings (*N* = 550)	Two-factor model (original)	.12	.89	.87	–	–	Cronbach’s α (.91); convergent, criterion, & known-groups validity
		Two-factor model (alternative)	.073	.81	.81

The Turkish version of the WHOQOL-AGE was also explored. Findings revealed an alternative two-factor model with different item compositions showed slightly better goodness-of-fit values than the original scale (Özcan & Eser 2020). However, both models showed poor comparative fit index (CFI) and Tucker-Lewis (TLI) values (< 0.90). It is recommended that a broad range of fit indices (e.g., predictive and absolute fit) and assessment of standardized loadings should be used (Morrison et al. 2017).

Current literature indicated that the psychometric properties of the WHOQOL-AGE have mostly been validated in Europe, while the investigation in Taiwan validated a translated version and did not include external criteria assessment (e.g., concurrent/divergent validity). Cultural and linguistic differences may cause translated questionnaires to have different psychometric properties than the original, so the properties of the original English version need to be examined in an Asian context. Furthermore, the WHOQOL-AGE (Caballero et al. 2013) has been used to assess overall QOL among older adults in Singapore (Shorey et al. 2021; Siew et al. 2021). It is timely that the suitability of this tool is formally established for general use in Singapore. This will deepen the understanding of the WHOQOL-AGE factor structure and accumulate further psychometric evidence for QOL literature. The validation of the WHOQOL-AGE would add evidence that the interpretation of scores is valid, encouraging professionals to continue using this tool to describe the QOL of the general population or specific groups in Singapore (e.g., people with health chronic conditions), and provide support for this tool to be used as a baseline and outcome measure to evaluate the effect of clinical and/or public health interventions on QOL. The validation of the WHOQOL-AGE would also allow the comparison of findings obtained in Singapore and Western countries. Therefore, the objectives of this study are to

1. verify the factor structure of the WHOQOL-AGE using another Asian sample,
2. evaluate the reliability and validity of the WHOQOL-AGE.

## Methods

### Design and sampling

This study was cross-sectional and used data collected through the Community Health and Intergenerational (CHI) study in Singapore between 2018 and 2021 (Lee et al. 2020). The CHI study sought to investigate vulnerability and protective factors of aging using a biopsychosocial framework. Eligible participants were older adults of any gender and ethnic group, residing in the community, with or without known medical conditions, and aged between 60 and 91 years. Convenience and snowball sampling were employed. Older adults were recruited via word of mouth, advertisement flyers at community centers and door-to-door house visits conducted by research assistants and nurses. Specifically, participants who lived within a 10 km radius from the research site (situated in the central-west region of Singapore) were recruited. The Department of Statistics of Singapore (2017) verified that over 3000 older adults resided in the Anak Bukit Area (i.e., subzone of central west district) of Singapore, where the research site was located. The present study was part of the larger CHI study and used an overall sample of 593 participants who responded to questionnaires in English (refer to Table 2 for participants’ characteristics). This study was approved by the Ethics Committee of the National University of [Singapore].

**Table 2:** Participants’ characteristics

Characteristics	Total sample (*N* = 593)
Age (years)	
Mean (*SD*)	67.19 (5.70)
Range	60–91
Gender, n (%)	
Male	239 (40.30)
Female	354 (59.70)
Marital Status, *n* (%)	
Single	84 (14.20)
Married	438 (73.90)
Widowed	46 (7.80)
Divorced/Separated	25 (4.20)
Ethnicity, *n* (%)	
Chinese	560 (94.40)
Malay	3 (0.50)
Indian	21 (3.50)
Others	9 (1.50)
Education (years)	
Mean (*SD*)	14.28 (3.49)
Range	2–27.50
Monthly Household income, *n* (%)	
No Income	56 (9.40)
Below $2000	151 (25.50)
$2000-$4999	145 (24.50)
$5000-$7999	64 (10.80)
$8000–10999	59 (9.90)
Above $11,000	118 (19.90)
Living Arrangements, *n* (%)	
Living alone	92 (15.50)
Living with others	501 (84.50)
Cognitive Status,* n* (%)	
Healthy	466 (78.40)
With cognitive impairment	127 (21.60)
Presence of Chronic Medical Condition, *n* (%)	
Yes	495 (83.50)
No	98 (16.50)

#### Sociodemographic information

Sociodemographic information included age, years of schooling, marital status divided into four categories (single, married, widowed, divorced/separated), gender (male or female), household income level grouped into six categories (no income, below $2000, $2000–$4999, $5000–$7999, $8000–$10,999 or above $11,000) and living arrangements grouped into living alone or living with others. Participants were also asked to indicate yes or no if they had been diagnosed with the following conditions: hypertension, hyperlipidemia, diabetes, stroke, heart conditions, asthma, kidney, chronic obstructive lung disease, tuberculosis, arthritis, osteoporosis, neurodegenerative disorders, cancer, anxiety/depression and thyroid problems. Participants were classified as having a chronic medical condition if they had at least one of the conditions listed above. Similar to previous studies (Klainin-Yobas et al. 2019; Yu et al. 2020), participants’ scores from a range of cognitive tests were evaluated by a panel of psychiatrists and their cognitive status was subsequently categorized as healthy or with cognitive impairment. The panel used Petersen’s (2004) criteria for mild cognitive impairment and assessed subjective cognitive complaints (via Clinical Dementia Rating), presence of objective cognitive impairment, as well as preserved functional independence.

#### WHOQOL-AGE (Caballero et al. 2013)

The WHOQOL-AGE contains 13 items and assesses general QOL in older adults. Scores range from 13 to 65 and items are rated using a 5-point Likert scale with two types of response format. For instance, items Q1–Q8 responses were classified as bipolar (e.g., very bad to very good), while items Q9–Q13 responses were classified as unipolar (e.g., not at all to completely). Higher scores suggest better QOL. The scale was found to have high internal consistency (e.g., Cronbach’s α values ranging from 0.84 to 0.91), good convergent, discriminant and criterion-related validity (Caballero et al. 2013; Santos et al. 2018; Özcan and Eser 2020).

#### Satisfaction with life scale (Diener et al. 1985)

The SWLS is a brief 5-item scale that assesses global life satisfaction rated on a 1 (Strongly Disagree) to 7 (Strongly Agree) point Likert scale. Scores range from 5 to 35, with higher scores indicating higher life satisfaction. The SWLS has demonstrated strong internal reliability with Cronbach’s α = 0.88 (Park et al. 2009; Vera-Villarroel et al. 2012) and test–retest reliability, *r* = 0.82 (Diener et al. 1985). Moreover, the SWLS has been found to positively correlate with QOL (*r* = 0.49) using the WHOQOL-BREF (Vera-Villarroel et al. 2012).

#### Friendship Scale (Hawthorne 2006)

The 6-item Friendship Scale measures social connectedness and is scored on a 0 (not at all) to 4 (almost always) scale, with scores ranging from 0 to 24. Higher scores indicated higher levels of social connectedness, while lower scores assessed levels of social isolation. It was found to have good internal reliability (Cronbach’s α = 0.83) and good concurrent and discriminant validity (Hawthorne 2008).

#### Compassion (Martins et al. 2013)

The 10-item Compassion Scale is used to measure compassion toward others across five domains and has an acceptable internal consistency of Cronbach’s α value of 0.82 (Martins et al. 2013). It uses a 7-point Likert scale with scores ranging from 7 to 70, and higher scores suggest greater level of compassion.

### Procedure

Participants were informed of the purpose of the research, procedures and potential risks involved. Thereafter, written consent was obtained. Participants were interviewed by trained research assistants and nurses. Interviews were conducted at the research site or in the participants’ homes. As part of the CHI study, questionnaires and assessments were collected over six separate visits, with each lasting approximately 1–2 h. Participants in this study completed the sociodemographic questionnaire in the first visit. Thereafter, participants completed the WHOQOL-AGE, SWLS, Friendship Scale and Compassion Scale during their second visit. Participants were offered a total of up to SGD$50 as a token of appreciation. The study procedure has been explained in more detail in a prior publication (Lee et al. 2020).

### Statistical analysis

The tenability of the hypothesized bifactor structure of the WHOQOL-AGE was investigated through CFA. CFA was used to compare the data against three structural models (refer to Fig. 1) and to explore whether the data obtained in Singapore fitted the bifactor model as found in previous studies (Lin et al. 2020; Santos et al. 2018). The three models included the original two-factor model (Model 1) made up of one second-order and two first-order factors (Caballero et al. 2013), a bifactor model (Model 2) and another two-factor model (Model 3) that was made up of two-correlated factors, both proposed by Santos and colleagues (Santos et al. 2018). The three models were compared using the χ^2^ difference test, whereby a model that had a significantly lower χ^2^ indicated a better fit (Lin et al. 2020). Although nonsignificant χ^2^ values indicate a good fit, the χ^2^ statistic is known to be sensitive to sample size and might be inflated (significant) when sample sizes are large (Schreiber et al. 2006; Burnham and Anderson 2002). Model fit was also assessed using goodness-of-fit indices, whereby adequate cutoff values were indicated by root-mean-square error of approximation (RMSEA) values < 0.08 (Browne and Cudeck 1992), comparative fit index (CFI) values > 0.90 (Bentler 1990), Standardized Root-Mean-Square Residual (SRMR) values < 0.08 (Hu and Bentler 1999), Tucker-Lewis Index (TLI) values > 0.90 (Bentler 1990) and the lowest Akaike Information Criterion (AIC) value (Schermelleh-Engel et al. 2003). Measurement quality of WHOQOL-AGE was further assessed by the magnitude of standardized loadings (> 0.40) between each latent construct and its manifest variables (Stevens 1996). Cronbach’s α coefficients were calculated to appraise the internal consistency of the WHOQOL-AGE. Convergent validity was examined using Spearman’s rho correlations (*r*_*s*_), while discriminant validity was evaluated by a Pearson’s correlation (*r*). Correlation coefficients ≥ 0.30 were considered evidence of convergent validity and lower correlation coefficients (*r* < 0.30) as evidence of discriminant validity (Kaplan and Saccuzzo 2017). Known-group validity was tested using a Student’s independent *t* test that compared WHOQOL-AGE scores between people with and without chronic medical conditions according to previous research (Caballero et al. 2013). Significant levels were set to an α level of 0.05. All statistical analyses were conducted using R software (R-4.1.1). CFA was conducted using the *lavaan* package (Rosseel 2012).

## Results

### WHOQOL-AGE factor structure

The distribution of scores for each item of the WHOQOL-AGE was normally distributed, whereby skewness values ranged between − 1.01 and − 0.29, and kurtosis values ranged between 0.62 and 2.82 (Kline 2011). CFA analyses and χ^2^ difference test were conducted to compare the data against three structural models (see to Fig. 1) that were identified in previous research (Caballero et al. 2013; Lin et al. 2020; Santos et al. 2018). All three proposed models had significant χ^2^ (*p* < 0.001). Among the proposed models, the data fit the hypothesized bifactor model (Model 2) the best. Goodness-of-fit indices obtained in each model are shown in Table 3. Results from the χ^2^ difference test to compare all three models also indicated that Model 2 significantly outperformed Models 1 (*p* < 0.001) and 3 (*p* < 0.001).

**Table 3:** Structural model comparisons (N = 593)

Models	χ^2^ (df)	RMSEA	RMSEA 90% CI		CFI	SRMR	AIC	TLI
			Lower	Upper				
Model 1^a^	268.93 (63)*	.074	.065	.083	.915	.052	12,869.158	.895
Model 2^b^	164.43 (52)*	.060	.050	.071	.954	.037	12,786.659	.931
Model 3^c^	308.89 (64)*	.080	.071	.089	.899	.057	12,907.114	.877

^a^Model 1 is the original two-factor model proposed by Caballero et al. (2013); items Q1-Q8 in factor 1; items 1, 9 -13 in factor 2

^b^Model 2 is the bifactor model proposed by Santos et al. (2018); items Q1-Q8 in factor 1; items Q9-Q13 in factor 2; all items embedded in an additional construct of QOL

^c^Model 3 is the two-factor model suggested by Santos et al. (2018); items Q1–Q8 in the factor 1; items Q9–Q13 in the factor 2; correlation between factor 1 and 2

^*^Model achieving significant levels at *p* < 0.001

**Fig. 2:**
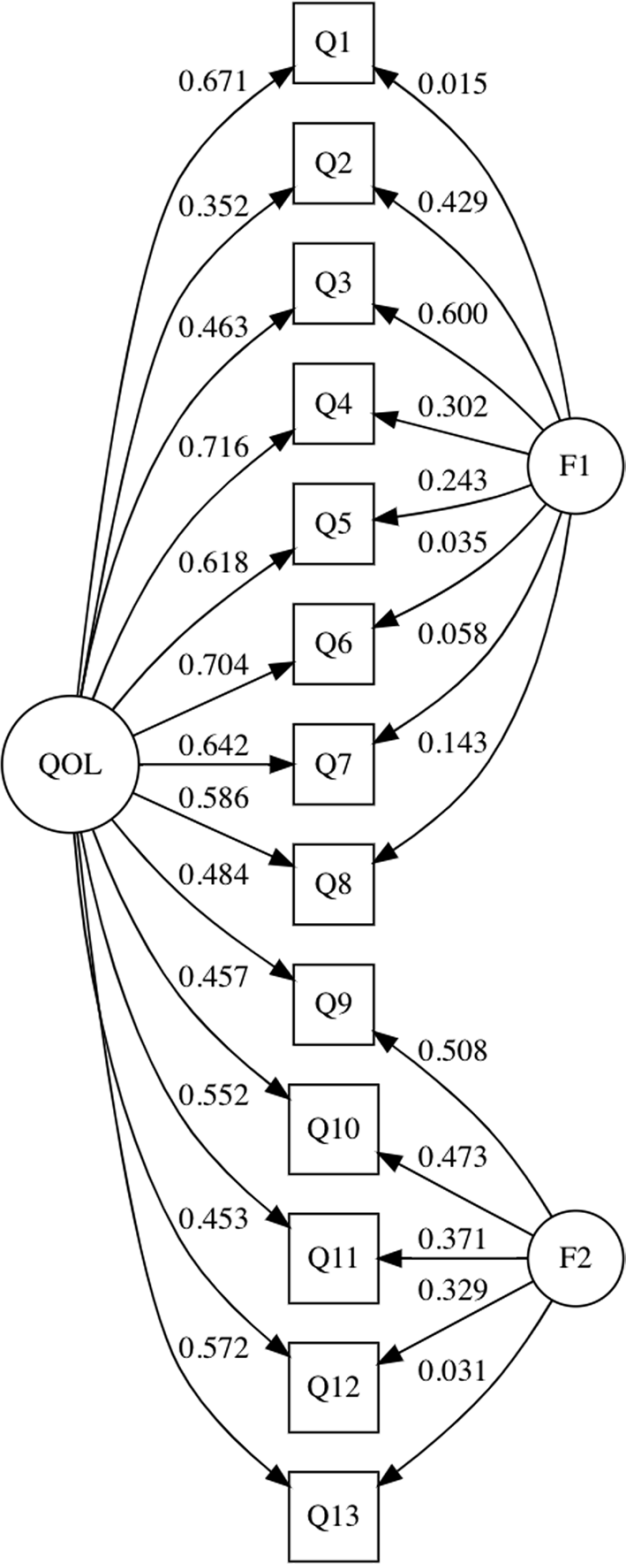
Bifactor model and its standardized regression coefficients

Model 2 (bifactor model) comprised a general QOL factor and two latent factors (e.g., F1: bipolar and F2: unipolar response scales). In Model 2, the standardized regression coefficients (i.e., factor loadings) of the general factor QOL were significant, *p* < 0.05 (see Fig. 2). In the bifactor model, all 13 items are associated with a general factor (loading: 0.352–0.716) to a much higher degree than with the two latent factors (loadings: 0.015–0.600). The general factor, which can be interpreted in terms of general QOL, is thus shown to be the dominant source of the item variances.

In Model 1, first-order standardized regression coefficients were significant (*p* < 0.001), and the factor loadings of items 1 to 8 on Factor 1 ranged from 0.254 to 0.791, while the factor loadings of item 1 and items 9 to 13 on Factor 2 ranged from 0.458 to 0.685. The second-order standardized loadings of Factor 1 and 2 on general QOL were both 0.865. In Model 3, the first-order factor loadings of Model 3 were significant (*p* < 0.001), whereby factor loadings of items 1 to 8 on Factor 1 (bipolar response scale) ranged from 0.447 to 0.778 and factor loading of items 9 to 13 on Factor 2 (unipolar response scale) ranged from 0.502 to 0.678.

### Internal reliability

Model 2 was used to test for internal consistency of the WHOQOL-AGE. Adequate Cronbach’s alpha values were found for each of the two latent factors (Cronbach’s *α* coefficient = 0.83 for Factor 1, Cronbach’s *α* = 0.73 for Factor 2) and the entire WHOQOL-AGE scale (Cronbach’s *α* coefficient was 0.86), indicating that all domains were consistent and within acceptable ranges (Cohen 1988).

### Convergent validity

To assess convergent validity of the WHOQOL-AGE, it was hypothesized that there would be medium-to-large positive correlation coefficients between QOL scores and life satisfaction (measured with the SWLS) as well as social connectedness (as assessed by the FS). Spearman’s correlation analyses revealed a large and statistically significant positive correlation between scores on the WHOQOL-AGE and SWLS scores, *r*_*s*_(591) = 0.54, *p* < 0.001, and a moderate and significant positive correlation between scores for the WHOQOL-AGE and social connectedness, *r*_*s*_(591) = 0.33, *p* < 0.001, indicating good convergent validity.

### Discriminant validity

To assess discriminant validity, it was hypothesized that there would be low correlation coefficients (*r* < 0.30) between QOL scores and scores from unrelated constructs such as compassion (measured with the Compassion Scale). A Pearson’s correlation analysis revealed a statistically significant low and positive correlation between QOL scores and level of compassion, *r*(591) = 0.19, *p* < 0.001.

### Known-groups Validity

Known-groups validity of WHOQOL-AGE was assessed by an independent *t* test, whereby the WHOQOL-AGE score was compared between participants with and without chronic medical conditions. Similar to previous research (Caballero et al. 2013), it was hypothesized that participants without chronic conditions would have higher QOL scores than those with at least one chronic condition. Results indicated that participants without chronic conditions had significantly higher QOL scores than participants with chronic condition, *t*(591) = 2.82,* p* < 0.01, Cohen’s *d* = 0.31.

## Discussion

The present study verified the factor structure of the WHOQOL-AGE using a Singapore sample and evaluated the scale’s reliability and validity. It is important to ensure that the original (English) version of the WHOQOL-AGE’s factor structure can be applied in Singapore. CFA findings revealed that the bifactor model with one general QOL factor and two specific group factors achieved acceptable fit and outperformed both the original second-order factor model and the two-correlated factor model. The bifactor model found in this study provided goodness-of-fit indices such as RMSEA, CFI, SRMR and TLI values that were consistent with a model of QOL previously found in Western (Santos et al. 2018) and Asian (Lin et al. 2020) populations. Findings suggest that the global concept of QOL in older adults in Singapore may be similar to the QOL proposed by WHO (Power et al. 2005; Skevington et al. 2004), whereby the structure of the QOL in Singapore seems to comprise a general component of QOL (items related to psychological, physical, social, sensory, environmental, autonomy and intimacy) and two components related to the questionnaire response scales. Moreover, previous studies using other WHOQOL measures in Singapore demonstrated sound psychometric properties (Suárez et al. 2018).

Factor loadings obtained in the study were similar to Santos et al.’s (2018) study, whereby most of the WHOQOL-AGE items displayed significant factor loadings for the general factor (QOL) as compared to the loadings for the two group factors (bipolar and unipolar response scale). In our community sample, the WHOQOL-AGE appears to capture well the general dimension of QOL. Moreover, it was argued that higher loadings on the general factor indicate that items primarily represent the general QOL construct and suggest against computing the subscale scores (Reise et al. 2010). Inspection of standardized factor loadings in this study also showed that Q2 on sensory abilities made the lowest contribution (below 0.40) to overall QOL. One possible reason could be due to the lack of sensory difficulties experienced by the participants in the present study (e.g., majority in their 60 s), as sensory problems are more prevalent in older adults over 80 years old (Homans et al. 2017) and impede functional independence and QOL with those advanced in age (Cimarolli & Jopp 2014). Additionally, all other items contributed significantly to overall QOL, mirroring previous findings (Lin et al. 2020; Liu et al. 2013), and suggest the importance of various aspects of well-being for overall QOL in older adults. Taken together, factor loadings on the general factor found in this study were generally acceptable, above 0.40 (Brown 2015), suggesting that all 13 items of the WHOQOL-AGE add valuable information to the global QOL.

The pattern of results also showed several items where the loadings were very high on the general and low on the group factor (< 0.10) such as Q1, Q6 and Q7 on Factor 1 (bipolar response scale) as well as Q13 on Factor 2 (unipolar response scale). It is possible that participants may have interpreted and rated Q1 different from the rest of items on Factor 1 due to differently worded bipolar response scales used (e.g., bad vs dissatisfied and good vs satisfied). It is suggested to use the same bipolar response scale and replace Q1 with “How satisfied are you with your quality of life?” to correspond with the rest of the items on Factor 1. Findings are also consistent with literature on QOL (Liu et al. 2013; Suárez et al. 2018) whereby some aspects seemed to contribute most to QOL than others (e.g., self-esteem, personal relationships). Also, Q13 a question on intimacy showed low loadings on Factor 2 which could be due to differently worded unipolar response scales (e.g., completely and an extreme amount) and culture as older adults are sensitive to topics about intimacy and may not express their true feelings when interviewed (Wang et al. 2006). Similarly, it is suggested that the same unipolar response scale is used and replace Q13 with “To what extent are you satisfied with your intimate relationships in your life?”. Future studies using exploratory factor analysis could be conducted to assess whether replacements could improve factor loadings on these items. Future research using exploratory factor analysis and CFA could explore revising low factor loading items to improve factor loadings.

The WHOQOL-AGE showed good internal consistency, consistent with the original development papers and previous two validation studies (Caballero et al. 2013; Lin et al. 2020; Santos et al. 2018). Convergent validity was demonstrated by moderate-to-large positive correlations between QOL and related constructs (e.g., satisfaction with life and social connectedness). This was in line with previous research (Dorji et al. 2017; Mei et al. 2021; Yang and Srinivasan 2016). The current findings also provide preliminary evidence of discriminant validity for the WHOQOL-AGE, demonstrated by low correlations between QOL and an unrelated construct (e.g., compassion) (Campbell & Fiske 1959). Consistent with the literature (Caballero et al. 2013; Özcan & Eser 2020), the WHOQOL-AGE discriminated between healthy individuals and individuals with at least one chronic medical condition, showing adequate known-groups validity, although the effect size of the difference between groups was relatively small (Cohen’s *d* = 0.31) as compared to previous studies (Caballero et al. 2013; Özcan & Eser 2020); one possible reason for such small effect size could be that the current sample was relatively younger (*M*_age_ = 67.19 years) and more physically independent as compared to those previous studies, and their chronic condition may still be in the early stages to influence their QOL. Future studies may further investigate whether the WHOQOL-AGE applied in Singapore is sensitive in detecting intervention effects and other variables (e.g., dependency) that were previously found to decrease QOL in older adults (Lobo et al. 2014; Tobiasz-Adamczyk et al. 2017).

It needs to be acknowledged that participants in this study were predominantly of Chinese descent (94.3%) community-dwelling older adults living in the central-west region of Singapore. This was similar to the ethnic distribution reported by the Department of Statistics, Singapore (2017), for the Anak Bukit Area of Singapore. However, the ethnic distribution of the older adult sample in this study (see Table 2) was slightly different from the total ethnic distribution of older adults in Singapore, whereby 74.07% residents were Chinese, 13.36%% were Malay, 9.23% were Indians, and 3.33% belonged to the others group (Department of Statistics 2010). While findings can be generalized to neighborhoods with similar ethnic proportions, it may be difficult to generalize to the general older adult population in Singapore, or clinical populations. In addition, the present study did not assess multigroup invariance and test–retest reliability of the WHOQOL-AGE; hence, further support is needed to evaluate its reproducibility, stability and construct validity. Future research could further conduct measurement invariance testing across different conditions as previously invariance was supported in Taiwan on across gender, education levels and living settings (Lin et al. 2020). As only the English version of WHOQOL-AGE was used in this study, future research could test for structural invariance across language and evaluate the validity of translated versions.

In conclusion, the present study demonstrated promising psychometric properties of the WHOQOL-AGE using a Singapore older adult sample. Findings from this study supplement current literature as the data fitted a bifactor model and showed adequate internal consistency, convergent and discriminant validity, and known groups validity. WHOQOL-AGE may provide healthcare professional and researchers a valid tool to assess QOL for older adults, especially in community settings or when time is limited. With further research, the WHOQOL-AGE can be used to evaluate intervention effects, assess community needs and inform treatment planning in Singapore and other Asian populations.
